# Pitfalls of invasive blood pressure monitoring using the caudal ventral artery in rats

**DOI:** 10.1038/srep41907

**Published:** 2017-02-13

**Authors:** Hiroki Ohta, Takao Ohki, Yuji Kanaoka, Makoto Koizumi, Hirotaka J. Okano

**Affiliations:** 1 Division of Regenerative Medicine, Research Center for Medical Sciences, the Jikei University School of Medicine, 3-25-8 Nishi-shinbashi, Minato-ku, Tokyo 105-8461, Japan; 2 Division of Vascular Surgery, Department of Surgery, the Jikei University School of Medicine, 3-25-8 Nishi-shinbashi, Minato-ku, Tokyo 105-8461, Japan; 3 Laboratory Animal Facilities, Research Center for Medical Sciences, the Jikei University School of Medicine, 3-25-8 Nishi-shinbashi, Minato-ku, Tokyo 105-8461, Japan

## Abstract

During rodent experiments, the caudal ventral artery (CVA) is useful for blood pressure (BP) measurement. However, CVA measurements may not reflect the true BP. This study was performed to verify the site-specific accuracy of invasive arterial BP monitoring during surgery in rats. Invasive arterial BP was simultaneously measured in rats via the CVA and the common carotid artery (CCA). The BP values were analysed while the rats were subjected to cooling of the head or tail. Additionally, the rats underwent digital subtraction angiography and histological examination of these arteries. The pressure difference was more significant in the tail cooling group than in the head cooling group. Digital subtraction angiography revealed that angiospasms occurred more frequently in the CVA than in the CCA upon cooling. This phenomenon was supported by histological analysis, which showed that the tunica media area was significantly larger in the CVA than in the CCA. CVA pressure is susceptible to environmental changes and may not accurately reflect the true BP without a strictly controlled laboratory environment. Therefore, understanding the pitfalls of this method is necessary to avoid cooling of the tail during BP measurement.

In humans, monitoring of a patient under general anaesthesia is an essential and common practice. Intraoperative monitoring includes measurement of the heart rate, blood pressure (BP), body temperature, oxygen saturation (SpO_2_), end tidal carboxylate concentration (ETCO_2_), and anaesthesia depth. These parameters are routinely monitored to ensure safety during surgery. This monitoring is particularly essential during bleeding or unstable circulatory dynamics^1^.

Intraoperative monitoring, however, has not been comprehensively performed in animals. In the animal experimentation field, intraprocedural death can occur due to neglect in BP monitoring. The perfusion of each organ in the body changes with the anaesthetic dosage, and systemic reactions are likely to influence laboratory findings. In humans, invasive arterial BP (ABP) measurement is fundamental during a critical state, especially during cardiovascular surgery and when bleeding is expected^2^. Similarly, researchers need to assess the haemodynamics during surgical treatment of animals and to regulate the anaesthesia depth; they also need to administer cardiovascular agents and provide fluid replacement. Preventing unplanned intraoperative death is also very important from an animal welfare perspective^3^.

Invasive ABP measurement requires complicated procedures to initiate the measurement, which is a disadvantage. Although wireless measurement devices via radiotelemetry are useful for the invasive monitoring of ABP, these devices are too expensive to employ for routine intraoperative monitoring^4^,^5^. Telemetry is used to observe BP changes in a freely moving animal with a buried BP measurement catheter. The survival of the animal is not influenced even if the blood flow of the tail is lost as a consequence of BP measurement via the caudal ventral artery (CVA). However, the influence of peripheral reflection waves can be significant because the vessel is far from the heart and small in calibre.

Peripheral BP measurements have been reported to be higher than central BP measurements^6^,^7^. However, our experimental data showed that the systolic pressure of the CCA was higher than the systolic pressure of the CVA. Moreover, the cannula in the CVA can be easily dislodged and is difficult to fix. Furthermore, accurate measurement of the BP using a peripheral artery may be difficult when the body temperature varies or if the animal has peripheral circulatory dysfunction.

Therefore, the accuracy of peripheral ABP monitoring may be questioned. To the best of our knowledge, few attempts have been made to invasively monitor the BP of the CVA and common carotid artery (CCA) simultaneously in rats. Thus, we performed invasive BP measurements at these two sites in rats under various conditions to verify the accuracy of ABP monitoring.

We conducted the present study to verify the usefulness of CCA ABP intraoperative monitoring in small animals. Specifically, we investigated whether simultaneous invasive ABP measurements in the CVA and CCA in rats yielded the same results under various conditions.

## Results

### BP monitoring equivalent to human intraoperative monitoring

In this study, the BP (total measurements of 851 pairs) was measured simultaneously in the CCA and the CVA in a single animal. The results were compared for measurement accuracy and to determine whether environmental influences caused site-specific pressure differences.

**Figure 1:**
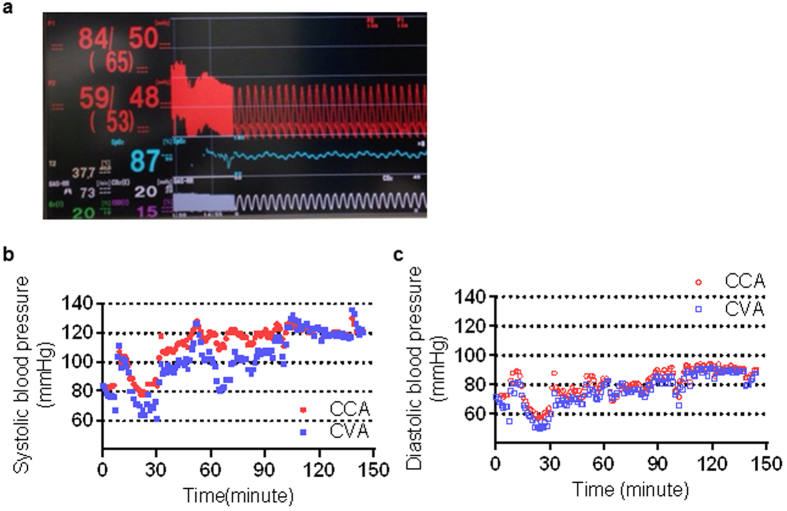
The systolic pressure of the caudal ventral artery does not reflect the common carotid artery. (**a**) Actual monitor screen displaying common carotid artery (CCA) pressure (red characters, “84/50”), caudal ventral artery (CVA) pressure (red characters, “59/48”), body temperature (white characters, “37.7”), SpO_2_ (blue characters, “87”), anaesthetic concentration (purple characters, “1.5”), EtCO_2_ concentration (white characters, “20”), and respiration rate (white characters, “73”). A difference in systolic blood pressure (BP) was observed between the CCA and CVA. (**b**,**c**) Correlation between the simultaneously recorded invasive CCA pressure (red dots) and invasive CVA pressure (blue dots) in the same animal. (b: systolic blood pressure, c: diastolic blood pressure).

Some parameters (ABP, body temperature, SpO_2_, ETCO_2_, and anaesthesia depth) were accurately monitored (Fig. 1a). Despite various attempts to noninvasively measure BP in the upper limbs, lower limbs, and tail, these measurements were difficult to obtain. Simultaneous measurements of CCA and CVA pressure obtained in the same animal were analysed (systolic BP: Fig. 1b, diastolic BP: Fig. 1c). The systolic CCA pressure was compared with the simultaneously measured systolic CVA pressure (Table 1). Four situations were established to compare the site-specific BP measurements with special attention paid to head or tail warming/cooling. The temperatures in the head and tail were maintained in the control group (HwTw). The head was cooled using an ice pack while the tail temperature was maintained in the HcTw group (HcTw). In contrast, the head temperature was maintained while the tail was cooled using an ice pack in the HwTc group (HwTc). To simulate haemorrhagic shock, 2 mL of blood was withdrawn from the animals in the bleeding group (bleeding). These groups are defined in greater detail in later sections.

The average discrepancies within the HwTw, HcTw, HwTc, and bleeding group were 10.8 ± 7.4, 14.0 ± 13.7, 25.2 ± 20.2, and 22.9 ± 13.8 mmHg, respectively. These groups are defined in greater detail in later sections. The diastolic average discrepancy of the HwTw group was 8.88 ± 9.21 mmHg. Both results were higher in the actual measurements from the CCA site. The difference observed in the HwTw group was greater in the systolic BP than in the diastolic BP. Therefore, the systolic BP values were analysed in this study.

### Comparisons of BP discrepancies

**Figure 2:**
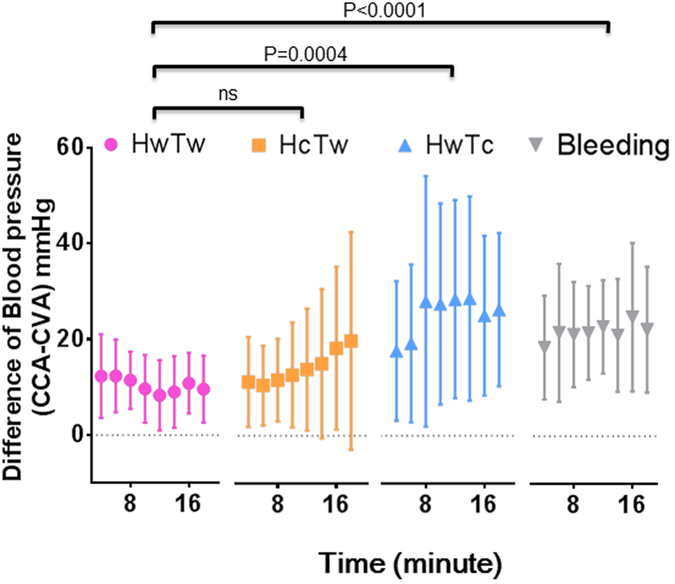
Comparison of systolic pressure discrepancies under various conditions. The differences in systolic ABP measured in the HwTw (both head and tail kept warm), HcTw (head cool, tail warm), HwTc (head warm, tail cool), and bleeding groups were 10.8 ± 7.42 mmHg, 14.0 ± 13.7 mmHg, 25.2 ± 20.2 mmHg, and 22.9 ± 13.8 mmHg, respectively. Although no significant difference was observed in the discrepancies between the HwTw and HcTw groups, a significant difference in pressure discrepancies was found between the HwTw and HwTc groups (P = 0.0004). Similarly, a significant difference was observed between the HwTw and bleeding groups (P < 0.0001). *x*-axis: time (minutes), *y*-axis: differences in systolic arterial blood pressure (mmHg), CCA: common carotid artery, CVA: caudal ventral artery.

**Figure 3:**
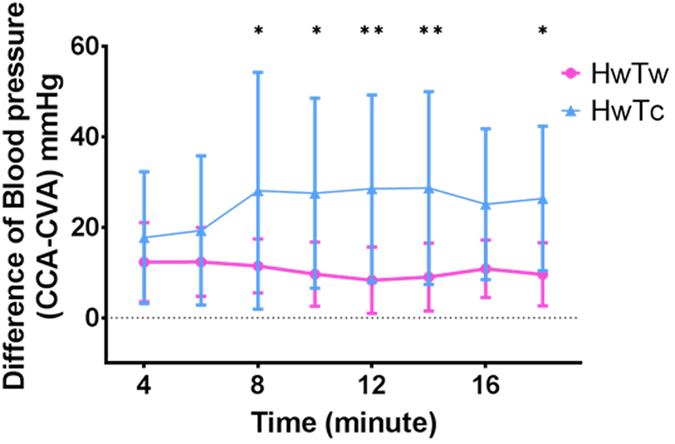
The systolic blood pressure difference was increased by cooling the tail of the rat. The graph shows the change over time under each condition. A significant blood pressure disparity was observed in the tail cooling group (HwTc) compared to the control group (HwTw). *(0.01 < P < 0.05) and **(P < 0.01) based on two-way ANOVA.

The time course showed differences in the systolic ABP between the HwTw, HcTw, HwTc, and bleeding groups (Fig. 2). The BP values were clearly different between the four situations. A significant difference in pressure discrepancy was observed between the HwTw and HwTc groups (*P* = 0.0004). The pressure discrepancy was larger in the bleeding group than in the normovolemic (HwTw) group (*P* < 0.0001). The pressure discrepancy was evaluated under haemorrhagic shock conditions because this condition could easily cause the peripheral arteries to collapse. The temperature maintained (HwTw) group, the head cooling (HcTw) group, and bleeding group showed less variation regardless of the time course (Supplemental Fig. 1). In contrast, the tail cooling (HwTc) group exhibited considerable variation (Fig. 3). Overall, significant differences were observed between the HwTw and HwTc groups at almost all time points. Tail cooling could also easily cause the tail artery to collapse.

### Causes of the BP discrepancies

**Figure 4:**
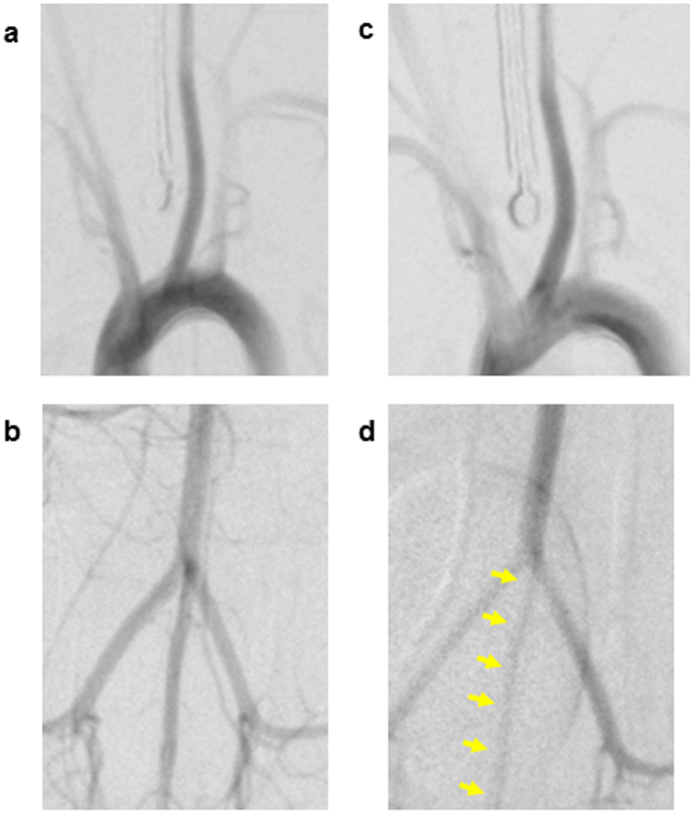
*In vivo* digital subtraction angiography showing a spastic caudal ventral artery after tail cooling in rats. (**a,b**) The left common carotid artery (**a**) and the caudal ventral artery (**b**) were visualised in the control (HwTw) group. (**c**) The left common carotid artery was not spastic in the head cooling (HcTw) group. (**d**) The caudal ventral artery was spastic (arrows) in the tail cooling (HwTc) group. Thus, tail cooling in rats can easily induce angiospasm of the tail artery.

**Figure 5:**
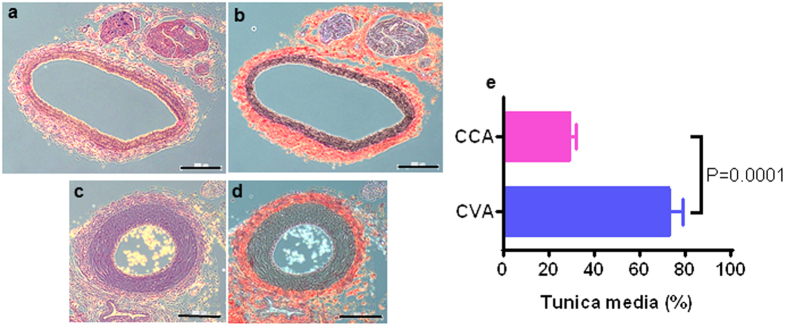
Thickened tunica media in the caudal ventral artery compared with the common carotid artery. (**a**,**b**) Histological sections of the common carotid artery (CCA) from a rat stained with haematoxylin and eosin **(a)** and Elastica van Gieson stain **(b)**. (**c,d**) Histological sections of the caudal ventral artery (CVA) from a rat stained with haematoxylin and eosin **(c)** and Elastica van Gieson stain **(d)**. The tunica media was considerably thicker in the CVA than in the CCA. Scale bar = 200 μm. (**e**) Comparison of the tunica media area ratio between the CCA and CVA. The tunica media area ratio in the CCA was significantly larger than in the CVA (29.84 ± 2.18% vs. 73.55 ± 5.27%, respectively, P = 0.0001). CCA: common carotid artery, CVA: caudal ventral artery.

The CCA and CVA of the rats were clearly detected by angiography. We focused on the HwTw and HwTc groups in this analysis. CCA spasms were absent in the control (HwTw) and the head cooling (HcTw) groups (Fig. 4a,b). In contrast, marked constriction of the CVA was observed in the tail cooling (HwTc) group (Fig. 4d) compared to the HwTw group (Fig. 4c). Histological evaluation of the CCA and CVA showed that the tunica media was thicker in the CVA than in the CCA (Fig. 5a–d). The ratio of the percent area of the tunica media to the cross-sectional area of the blood vessel was significantly larger in the CVA than in the CCA (73.55 ± 5.27% vs. 29.84 ± 2.18%, respectively; *P* = 0.0001; Fig. 5e).

## Discussion

Textbooks usually state that the peripheral systolic BP is higher than central BP in humans^6^,^7^. The peripheral ABP is also perceived as higher than the central ABP in rats^1^. However, our experimental data showed that the systolic pressure was higher in the CCA than in the CVA. When monitoring ABP, a difference reportedly exists between invasive CCA pressure and tail-cuff pressure. This experiment suggests that BP measurement in the CVA is not accurate.

The most commonly used form of BP monitoring in rats is non-invasive BP (NIBP) measurements, which are frequently performed with a cuff on the tail due to the fact that this method is simple and easily repeatable^8^. NIBP measurement in the CVA has been commonly used to assess BP in rodents because this method is both inexpensive and noninvasive^9^,^10^. However, this method has its limitations. CVA measurements have been suggested to not reflect the true BP^11^. The American Heart Association has recommended that animals be exposed to the BP measurement procedure daily for 7–14 days before beginning an experiment^3^. NIBP measurement also requires strict body temperature control, and efforts must be made to match the measurement conditions each time^12^. Additionally, because each measurement takes approximately 1–2 min, NIBP measurement is not suitable for monitoring instantaneous haemodynamic changes during surgery. Furthermore, preparation of the animal for BP measurement using a tail cuff requires both time and effort. Suitable methods of heating and restraint are essential for accurate BP measurements^13^. Moreover, similar to the “white coat” hypertension caused by transient stress in humans, the BP may increase from a similar stress response when the tail-cuff method is used in conscious animals^14^. As described above, NIBP measurement may seem easy, but maintenance of the correct environment is complicated and necessary when the measurements are performed in small animals to ensure the accuracy of the data. Additionally, NIBP measurements cannot measure the BP in real-time. Therefore, non-invasive monitoring during surgery in rats may be of questionable value.

The second standard method for BP monitoring in rats is ABP measurement. This method is useful for monitoring circulation in anesthetised animals^15^, but ABP monitoring is an invasive method that is difficult to perform in small animals^16^. Two ABP measurement methods are available: the telemetry method and the catheter method. The catheter method allows instantaneous and inexpensive ABP monitoring in animals during surgery^17^. This method is useful in this respect but also has some disadvantages. First, catheterisation is performed under general anaesthesia, which may lead to haemodynamic changes. Second, the researcher needs to be familiar with catheterisation procedures. The telemetry method is used to observe BP changes in freely moving animals after a BP monitor has been surgically implanted^4^,^5^. This procedure can be used after awakening from anaesthesia, but it is expensive and not a realistic method for intraoperative monitoring. Therefore, the catheter method is the most suitable for ABP monitoring during surgical procedures.

The BP varies with changes in circulatory dynamics; therefore, precise monitoring of the BP to avoid intraoperative death or shock is necessary if an experiment is susceptible to changes in haemodynamics. Similar to procedures in humans undergoing surgery, invasive ABP measurements are desirable in animal experiments to detect sudden changes in haemodynamics. The catheter method allows the BP to be measured with relatively inexpensive equipment and has the advantage of allowing the results to be displayed on a monitor in real time. Additionally, drug administration and fluid injection can be performed via the catheter used for the ABP measurements, thereby preventing unnecessary animal deaths. Indeed, the survival of the animal is not affected even if the tail is lost as a consequence of BP measurement via the CVA. The CVA is easy to approach, which makes the CVA a commonly used target blood vessel. However, the influence of peripheral reflection waves can be marked because the vessel is far from the heart. Peripheral BP measurements have been reported to be higher than central BP measurements. Moreover, fixing the cannula in the CVA is difficult and it can be prone to dislodging. Furthermore, accurate measurement of BP using a peripheral artery may be difficult when the body temperature varies or if the animal has peripheral circulatory dysfunction. Therefore, the accuracy of peripheral ABP monitoring may be questioned. To the best of our knowledge, few attempts have been made to invasively monitor the BP of the CVA and CCA simultaneously in rats. Therefore, we performed invasive BP measurements at these two sites in rats. We compared the pressure range at each site and determined the length of time before the BP changed in response to a change in body temperature.

Our study showed that the CVA BP was significantly reduced by cooling of the tail. In contrast, the CCA was stable after cooling of the head. These results suggest that measurement of CVA pressure may be inadequate for the precise assessment of intraoperative haemodynamics. Accordingly, we confirmed that the CVA underwent vasoconstriction in response to hypothermia of the tail. From the histological analysis, we can predict that occlusion of blood flow in the CVA is likely due to vasospasms. This result suggests the importance of maintaining the tail temperature during NIBP measurements of the tail to obtain accurate BP measurements.

Studies investigating haemodynamics using rodent models are often performed with non-invasive ABP measurements using a tail cuff. Our study shows that strict control of the tail temperature is necessary when the tail is used for BP monitoring. In contrast, an intraoperative CCA BP monitoring system can be used to accurately monitor haemodynamics in real-time, which may lead to safer surgeries. This monitoring approach may also prevent accidental death during experiments and thus is important for laboratory animal welfare. Because the BP is affected by multiple factors (e.g., operation type and duration, posture during operation, and anaesthesia depth), further studies are required.

As mentioned above, the CVA is useful for BP measurements in rodent experiments. However, CVA measurements may not reflect the true BP. This study was performed to verify the site-specific accuracy of invasive arterial BP monitoring during surgery in rats. We have clarified that vasospasms are a cause of inaccurate BP measurements. Furthermore, we report an example of vasospasms identified by angiography and demonstrate their cause based on a pathological analysis.

## Methods

### Animals

This study was approved by the Institutional Animal Care and Use Committee of the Jikei University School of Medicine (protocol number: 24-049C2). All procedures were conducted according to the Fundamental Guidelines for Proper Conduct of Animal Experiments and Related Activities in Academic Research Institutions issued by the Japanese Ministry of Education, Culture, Sports, Science and Technology. Male normotensive Sprague-Dawley rats (*n* = 10; 9–12 weeks of age; 276–499 g on the day of surgery) obtained from Nihon SLC (Japan SLC, Inc. Shizuoka, Japan) were housed in polycarbonate cages under temperature-controlled conditions (temperature: 24–25 °C; relative humidity: 50–60%) with a 12-h light–dark cycle. All rats had free access to water and pelleted food (CE-2, CLEA Japan, Inc., Tokyo, Japan). Each animal was maintained in a pain-free state during surgery by appropriately regulating the inhaled anaesthetic based on the CCA BP and heart rate. Animals were maintained at a body temperature of 36–39.5 °C during surgery.

### Anaesthesia and surgical procedure

Surgery was performed in the hybrid operating room with digital subtraction angiography (Artis Zee, Siemens, Germany) for animal experiments in the animal facilities of Jikei University School of Medicine. Anaesthesia was induced with a mixture of medetomidine hydrochloride (0.03 mg/mL), midazolam (0.4 mg/mL), and butorphanol tartrate (0.5 mg/mL) administered by intraperitoneal injection at 5 mL/kg^16^,^18^,^19^,^20^. Then, the rats were endotracheally intubated with a 16-gauge catheter (Terumo, Tokyo, Japan). After intubation, atipamezole hydrochloride (0.7 mg/kg) was intraperitoneally injected to reverse the effects of medetomidine. Anaesthesia was maintained through ventilation using an animal ventilator and 1–3% isoflurane after intubation. Monitoring of vital signs (BP, SpO_2_, and temperature) was performed in real-time using the Life Scope VS (Nihon Kohden, Tokyo, Japan; Fig. 1a). After ventilation and circulation were stabilised, the body temperature was maintained using a multipaneled heater (Vivaria, Osaka, Japan). The body temperature was measured with a small animal probe inserted into the oesophagus and was appropriately controlled at 36–39.5 °C.

Catheters for invasive ABP monitoring were inserted into the CVA and left CCA. First, the rat was placed in the supine position, and the tail skin was sterilised with povidone-iodine. Then, a 24-gauge catheter (Terumo, Tokyo, Japan) was aseptically inserted into the CVA to allow BP measurement. After backflow from the catheter was confirmed, the catheter was connected to the introducer of the arterial line. Visualisation of the arterial waveform on the monitor was confirmed, and the catheter was flushed with heparinised physiological saline.

A pillow was placed under the shoulders of the rat such that the neck was hyperextended, and the neck skin was sterilised with povidone-iodine. An incision was made in the midline of the neck. The incision was deepened through the avascular plane. The left CCA was exposed by peeling back superficial tissues to the left along the surface of the sternohyoid muscle. The sheath of the left CCA was blunt dissected to expose the CCA at the site where the vagus nerve passed laterally to the CCA. Then, 4–0 silk ligatures were passed around the proximal and distal portions of the CCA, and a 24-gauge catheter was inserted towards the proximal direction. After backflow from the catheter was confirmed, the catheter was connected to the introducer of the arterial line. After confirming a waveform appearance on the monitor, the catheter was flushed with heparinised physiological saline and fixed in place by silk ligature.

### Study protocol

The following data were recorded every 1 min: respiration rate, left CCA pressure (systolic, diastolic, and mean), CVA pressure (systolic, diastolic, and mean), body temperature, ETCO_2_, and anaesthesia depth. A device for SpO_2_ measurement was attached to the upper extremity of the rat. Invasive BP monitoring was simultaneously performed in the CVA and left CCA. After the body temperature of the rat was regulated to a normal level of 38.5 °C ± 0.5 °C, the ABP was measured at the CCA and the CVA. The temperature in the head and tail was maintained in this group (HwTw control group). Then, the head and neck were cooled using an ice pack above the subclavian bone while the tail temperature was maintained (HcTw group). Subsequent the head temperature was maintained while the tail was cooled (HwTc group). To simulate haemorrhagic shock, 2 mL of blood was withdrawn from the animals (the bleeding group). In each group, the BP was measured simultaneously at both the CCA and CVA sites. The BP values were measured every two minutes, and the mean values were used for the analysis.

Angiography of the CVA and CCA was performed in each group using digital subtraction angiography to evaluate the presence or absence of vasoconstriction. Angiography was performed to determine the cause of the pressure measurement discrepancy. The CCA and CVA were harvested after euthanasia by injection of sodium pentobarbital (200 mg/kg). These specimens were stained with haematoxylin and eosin and Elastica van Gieson stain, and histological examinations were performed using a light microscope.

### Exclusion criteria

Rats with any of the following values were excluded due to potential physiological dysfunctions or monitoring errors: systolic BP > 180 mmHg or <40 mmHg, pulse pressure <5 mmHg, body temperature <35 °C, and respiratory arrest.

### Statistical analysis

The data were analysed using PRISM ver. 7.0.1 (GraphPad Software, Inc., CA, USA). The results were expressed as the mean ± standard deviation. The data from the four treatment groups (HwTw, HcTw, HwTc, and bleeding group) were analysed using one-way ANOVA. The time-course analysis between each group was used for two-way ANOVA. To exclude the carry-over effect, the first and last data measurements were removed from the one-way and two-way ANOVA analyses. Student’s *t*-test was used for the comparisons between 2 groups in the histological examinations. Significant differences are indicated by p values in the figures.

## Additional Information

**How to cite this article**: Ohta, H. *et al*. Pitfalls of invasive blood pressure monitoring using the caudal ventral artery in rats. *Sci. Rep.* **7**, 41907; doi: 10.1038/srep41907 (2017).

**Publisher's note:** Springer Nature remains neutral with regard to jurisdictional claims in published maps and institutional affiliations.

## Supplementary Material

Supplemental Figure 1

## Figures and Tables

**Table 1:** Blood pressure measurements in the four treatment groups.

Time (minute)	2	4	6	8	10	12	14	16	18	20	Average
HwTw											
CCA	106.1 ± 36.7	109.2 ± 14.0	111.2 ± 16.0	111.3 ± 21.7	110.8 ± 20.4	109.0 ± 19.4	106.2 ± 20.8	103.3 ± 22.1	92.5 ± 21.4	100.3 ± 19.9	106.7 ± 19
CVA	91.4 ± 10.2	96.9 ± 12.9	98.8 ± 16.0	99.9 ± 22.5	101.2 ± 20.1	100.7 ± 18.4	97.2 ± 18.6	92.4 ± 20.5	90.5 ± 18.3	90.1 ± 17.8	95.89 ± 18
Difference	14.1 ± 8.8	12.3 ± 8.7	12.4 ± 7.6	11.5 ± 6.0	9.7 ± 7.1	8.3 ± 7.3	9.0 ± 7.5	10.9 ± 6.4	9.6 ± 7.0	10.2 ± 7.4	10.8 ± 7.4
HcTw											
CCA	101.3 ± 14.3	100.3 ± 13.5	102.1 ± 12.3	100.7 ± 12.5	100.1 ± 11.0	98.7 ± 10.7	99.3 ± 9.7	98.5 ± 12.4	100.6 ± 10.4	100.2 ± 8.6	100.2 ± 11.5
CVA	90.1 ± 10.7	89.0 ± 9.7	91.6 ± 6.5	89.0 ± 6.3	87.4 ± 6.5	84.8 ± 9.0	84.2 ± 10.5	80.2 ± 12.9	80.8 ± 16.1	83.1 ± 17.4	86.1 ± 11.4
Difference	11.3 ± 9.4	11.3 ± 9.4	10.5 ± 8.3	11.7 ± 8.6	12.7 ± 10.9	13.9 ± 12.8	15.1 ± 15.6	18.3 ± 17.0	19.8 ± 22.7	17.0 ± 17.3	14.0 ± 13.7
HwTc											
CCA	102.7 ± 12.8	102.9 ± 11.8	102.1 ± 12.1	99.4 ± 14.3	96.7 ± 14.8	94.9 ± 14.5	95.2 ± 13.5	95.6 ± 13.1	94.9 ± 14.0	93.9 ± 12.9	97.9 ± 13.5
CVA	83.6 ± 18.0	84.3 ± 16.9	82.2 ± 15.8	75.0 ± 21.0	69.2 ± 18.9	66.4 ± 18.4	66.5 ± 18.7	69.3 ± 16.6	67.4 ± 16.8	70.2 ± 14.4	73.6 ± 18.7
Difference	19.1 ± 15.3	17.7 ± 14.6	19.3 ± 16.5	28.1 ± 26.2	27.6 ± 21.0	28.6 ± 20.7	28.7 ± 21.3	25.1 ± 16.7	26.4 ± 16.0	33.1 ± 29.6	25.2 ± 20.2
Bleeding											
CCA	90.2 ± 17.2	83.4 ± 16.2	84.0 ± 16.6	90.3 ± 21.0	87.5 ± 17.9	85.5 ± 16.9	83.5 ± 15.6	88.9 ± 25.0	88.1 ± 22.3	86.4 ± 25.9	86.7 ± 19.3
CVA	60.6 ± 16.4	57.5 ± 11.0	58.7 ± 15.2	67.1 ± 25.0	65.7 ± 21.5	62.7 ± 19.0	61.8 ± 19.6	67.1 ± 28.5	68.3 ± 27.1	65.2 ± 28.8	63.4 ± 21.3
Difference	29.1 ± 24.1	18.5 ± 10.8	21.6 ± 14.4	21.2 ± 11.0	21.6 ± 9.8	22.8 ± 9.7	21.1 ± 11.8	24.9 ± 15.5	22.3 ± 13.1	25.0 ± 13.2	22.9 ± 13.8

The table shows the actual time course values obtained in the HwTw (both head and tail kept warm), HcTw (head cool, tail warm), HwTc (head warm, tail cool), and bleeding groups with the mean±SD (mmHg). The HwTw group exhibited the least variation in blood pressure among the groups.
