# Supplementary material for: Pitfalls of invasive blood pressure monitoring using the caudal ventral artery in rats
Source: Sci Rep. 2017 Feb 13;7:41907. doi: 10.1038/srep41907 (PMC5304151; doi:10.1038/srep41907)
Supplement: Supplemental Figure 1 [file srep41907-s1.pdf]

Pitfalls of invasive blood pressure monitoring using the caudal ventral artery in rats

Hiroki Ohta[1,2], Takao Ohki[2], Yuji Kanaoka[2], Makoto Koizumi[3], and Hirotaka J Okano[1]

Supplemental Figure 1

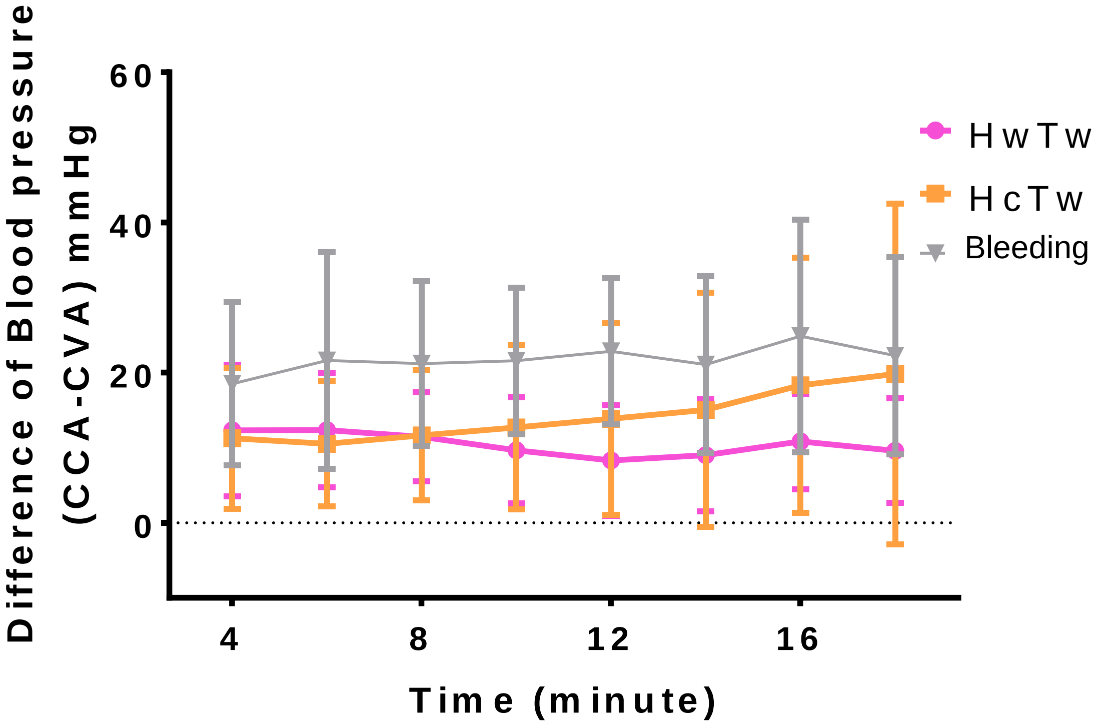

Supplemental Figure 1. No significant difference between HwTw, HcTw and bleeding groups
The graph shows the change over time under each condition. A significant blood pressure disparity was not observed in the head cooling group (HcTw) nor the bleeding group (Bleeding) compared to the control group (HwTw) based on two-way ANOVA.
